# The establishment of a fungal consortium in a new winery

**DOI:** 10.1038/s41598-020-64819-2

**Published:** 2020-05-14

**Authors:** Hany Abdo, Claudia Rita Catacchio, Mario Ventura, Pietro D’Addabbo, Hervé Alexandre, Michèle Guilloux-Bénatier, Sandrine Rousseaux

**Affiliations:** 1 Université Bourgogne Franche-Comté, AgroSup Dijon, PAM UMR A 02.102, F-21000 Dijon, France- IUVV Equipe VAlMiS, rue Claude Ladrey, BP 27877, 21078 Dijon, Cedex France; 2 Department of Biology, University of Bari, Bari, 70125 Italy

**Keywords:** Food microbiology, Fungal ecology, Microbial ecology

## Abstract

The biodiversity and evolution of fungal communities were monitored over a period of 3 vintages in a new winery. Samples were collected before grape receipt and 3 months after fermentation from 3 different wine related environments (WRE): floor, walls and equipment and analyzed using Illumina Mi-Seq. Genera of mold and filamentous fungi (294), non-enological (10) and wine-associated yeasts (25) were detected on all WREs before the arrival of the first harvest. Among them, genera like *Alternaria* and *Aureobasidium* persisted during two vintages. Therefore, these genera are not specific to winery environment and appear to be adapted to natural or anthropic environments due to their ubiquitous character. Some genera like *Candida* were also detected before the first harvest but only on one WREs, whereas, on the other WREs they were found after the harvest. The ubiquitous character and phenotypic traits of these fungal genera can explain their dynamics. After the first harvest and during 3 vintages the initial consortium was enriched by oenological genera like *Starmerella* introduced either by harvest or by potential transfers between the different WREs. However, these establishing genera, including *Saccharomyces*, do not appear to persist due to their low adaptation to the stressful conditions of winery environment.

## Introduction

Over the years, extensive research has been conducted on microbial biodiversity during the winemaking process. Microorganisms including filamentous fungi, bacteria and yeasts have been identified throughout the whole process using conventional culture-dependent techniques or molecular methods resulting in the description of this process as a true microbial ecosystem^1,2^. To understand the different roles of these microorganisms, especially yeasts, in wine fermentation and their impact on wine quality, in-depth knowledge of their population dynamics is needed. The development of recent technologies such as Next-Generation Sequencing (NGS) provided more complete understanding of the complexities of microbial communities^3,4^. These methods have uncovered that the microbial diversity is higher than expected^5,6^ and new microbial species (*Cytospora, Gigaspora, Naganishia, Rhodosporidiobolus, Vuilleminia*) have also been described^7–9^. Moreover, these methods made it possible to study diversity in more varied ecosystems like Wine-Related Environments (WREs: floor, walls and equipment)^10^. All these works described the presence of the same genera or species in different WREs and during the winemaking process, suggesting the persistence of these microorganisms during several vintages and their transfer between different WREs or between WREs and must or wine.

Intraspecific fingerprinting techniques were used to confirm the persistence and the transfer of microorganisms like *Saccharomyces cerevisiae*. Thus, techniques like mtDNA-RFLPs and interdelta PCR, were used to demonstrate the implantation and/or persistence of commercial strains of *S. cerevisiae* in must/wine and in WREs^11–14^. Indeed, a previous study showed that, although no longer used, commercial strains of *S. cerevisiae* can survive in the winery ecosystem for one year^12^. Concerning autochthonous *S. cerevisiae*, despite its low presence in the vineyard^15^, studies have shown that certain strains implicated in spontaneous fermentations originated from the winery environment^13,16,17^, or in some cases from the vineyard^17^. Moreover, implantation and persistence on the WREs and during the alcoholic fermentation (AF) were also highlighted for several non-*Saccharomyces* species^18,19^. Using FT-IR spectroscopy, the persistence of the species *Hanseniaspora guilliermondii* and *Hanseniaspora uvarum* during two vintages in the winery environment and their implantation in grape must have been previously demonstrated^19^.

All these findings highlighted: (i) the important role of WREs as an ecological niche for winery flora, and (ii) the implication of winery resident flora in the winemaking process.

From the beginning of the 21^st^ century, the number of new wine holdings has been increasing worldwide^20,21^. To our knowledge, only 3 studies were focused on yeast biodiversity in newly established wineries during several vintages^22–24^. But, none of them assessed the total fungal population (yeasts, mold and filamentous fungi) present in WREs and especially before the arrival of the first harvest. Moreover, the authors focused only on the diversity of the yeast population using a culture-dependent technique (mtDNA-RFLP) and on the effect of using commercial strains on the diversity of yeast populations found during the AF. Indeed, the results showed the capacity of starter strains to persist and become resident in the environment of new wineries^22,24^; however, no real evidence of their presence was proven in WREs.

Considering the growing interest in vinification with indigenous flora, the study of the evolution of microbial populations on the WREs of a new winery without using commercial strains is of high interest.

In this direction, in the present study we collected several samples from three different winery surfaces (floor, walls and equipment), for three consecutive vintages (2016, 2017 and 2018) and at separate time points (before grape harvest and 3 months after fermentation) and used NGS technologies to study the fungal biodiversity populations in the WREs of a new winery. We were able to study the microbial dynamics over time focusing on the contribution of fungal populations present on grapes and in must to WREs populations. We gained new insights on the capacities and mechanisms developed in the establishment or colonization of a fungal consortium in the winery environment.

## Results and Discussion

### Fungal status of the new winery before the arrival of the first harvest

**Figure 1:**
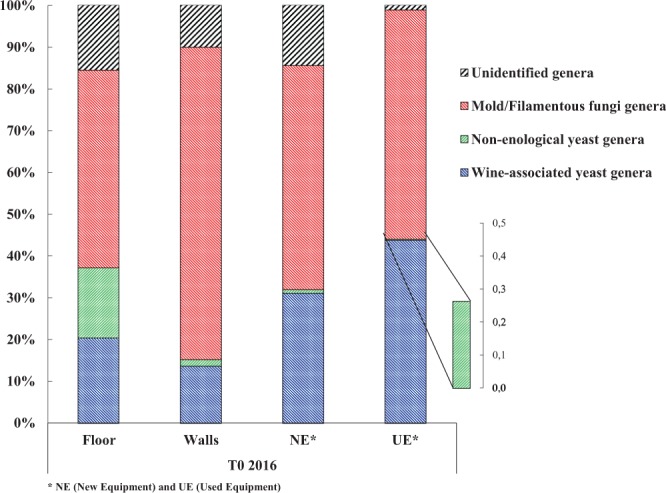
Percentages of the different fungal genera found on the different WREs of the new winery before the arrival of the first harvest (T0 2016).

The fungal diversity on the WREs of a new winery operating exclusively in spontaneous fermentation was studied to determine the fungal status of the winery before the arrival of the first harvest (T0 2016). Samples were collected from the floor, walls, new (NE) and used (UE) equipment. After Illumina sequencing, fungal OTUs were classified into 3 categories: (i) the mold and filamentous fungi genera, (ii) the yeast genera never described before in the winemaking process (named non-enological yeasts), and (iii) the yeast and yeast-like genera already described in the winemaking process (named wine-associated yeasts) (Fig. 1). Wine-associated yeasts include all the genera described in the literature in the vineyard, vine, grapes, must, wine and winery surfaces^25–29^.

Mold and filamentous fungi dominated the fungal genera identified on all the surfaces before the first harvest, with 294 different genera (Fig. 1 and Table 1). Yeast genera (non-enological and wine-associated) were identified before the arrival of any harvest in any of the WREs (Fig. 1 and Table 1) and a total of 35 different genera were found (Table 1). Wine-associated yeasts were identified on all the studied surfaces of the new winery and dominated the yeast population detected (Fig. 1 and Table 1). The identification of non-enological yeast genera may have resulted from the use of high-performance Illumina sequencing and the constant enrichment of the databases used^7,30^. The new winery surfaces (floor, walls and new equipment) showed a higher percentage (average 13%) of unidentified genera compared to the used equipment (1%) (Fig. 1).

**Table 1:** Number of identified fungal genera on the different WREs of the new winery before the arrival of the first harvest (T0 2016).

	Floor	Walls	NE*	UE*	All WREs
Wine-associated yeast genera	19	12	9	14	25
Non-enological yeast genera	5	7	5	5	10
**Total different yeast genera**	**24**	**19**	**14**	**19**	**35**
Mold & Filamentous Fungi genera	59	146	66	34	294
**Total different identified genera**	**83**	**165**	**80**	**53**	**329**

*NE (New Equipment) and UE (Used Equipment).

### Diversity and dynamics of mold and filamentous fungi genera

**Figure 2:**
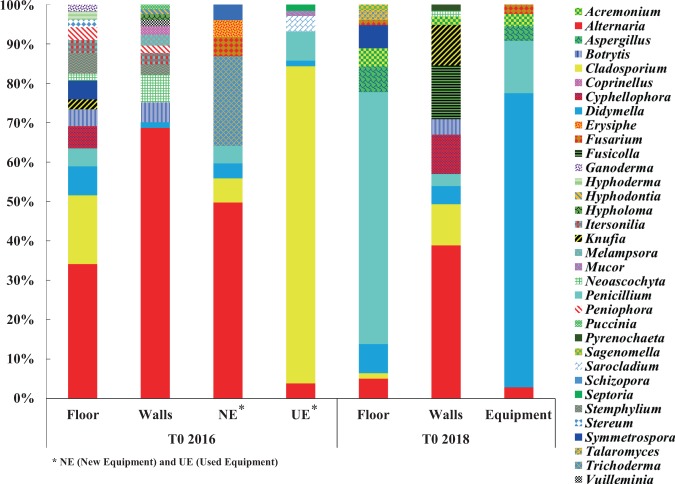
Relative abundance of the most represented mold and filamentous fungi genera identified on the different WREs prior to the 2016 and 2018 harvests. Represented genera are >1% relative abundance.

The different genera of mold and filamentous fungi identified at T0 2016 are represented using their relative abundances. Only the genera that represented >1% of relative abundance were selected (Fig. 2). Thus, a total of 32 mold and filamentous fungi genera among the 294 were identified on all the winery surfaces. At T0 2016, the taxonomic profiles observed on the floor, walls and the new equipment were quite close (with high representativeness of the genus *Alternaria*) compared to the used equipment which was dominated by the genus *Cladosporium* with the highest relative abundance (79%). These two genera had opposing relative abundances (a high abundance of *Alternaria* occurs with a low abundance of *Cladosporium* and vice-versa), suggesting potential interaction.

After two years of operations, a considerable modification of taxonomic profiles was observed, especially for the winery floor and equipment. These modifications resulted in a significant decrease in the total number of different identified genera (32 genera at T0 2016 and only 16 genera at T0 2018), as well as evolutions in the dominant genera (Fig. 2). So, the dominant genera *Alternaria* and *Cladosporium* described at T0 2016 were detected to a lesser extent at T0 2018, but persisted after 2 vintages. Their persistence throughout the whole study can be due to the presence of spores that might have been deposited from the surrounding atmosphere of the winery. Indeed, spores of *Alternaria* and *Cladosporium* are known to be widespread airborne spores^31^. Opposite abundances between the genera *Alternaria* and *Cladosporium*, observed at T0 2016, were also observed at T0 2018, suggesting again potential interaction (Fig. 2). After two years of operations, some genera became more widespread, like the genera *Penicillium* on the floor (57%) and *Didymella* on the equipment (68%). On the walls, *Alternaria* remained the most detected genus (35%), but new genera such as *Cladosporium, Cyphellophora, Fusicolla, Knufia* and *Penicillium* were also detected. The genus *Acremonium* was newly detected on all the WREs and the genus *Aspergillus* on the floor and equipment (Fig. 2).

Based on these results, implantation, colonization and persistence of some genera of mold and filamentous fungi were observed. For example: the genera *Alternaria* and *Didymella* present on all WREs between the vintages 2016 and 2018 can be considered as resident genera capable of persistence. After 2 years, genera *Cladosporium* and *Penicillium* are able to establish in all environments and persist. All these genera can therefore be qualified as ubiquitous genera. As for the genera *Acremonium* and *Aspergillus*, their implantation on environments might be linked to human activity and/or ventilation, which is an important factor of transfer of microorganisms between winery surfaces^32^. In addition, humidity and temperature described as two parameters influencing the growth of mold and filamentous fungi on surfaces like building material^33–35^ may also be factors influencing the establishment and persistence of the genera present on WREs.

### Diversity and dynamics of non-enological yeast genera

Ten different genera of non-enological yeasts were detected before the arrival of the first harvest on all WREs. The genera *Exophiala* and *Hannaella* were the only genera present on all the winery surfaces with the highest percentage on winery floor (25% and 10%, respectively) (Table 2). After the 2 genera mentioned above, the genus *Leucosporidium* is the one with the highest percentages on winery floor (9%) and on walls (5%). These three genera are usually found in natural ecosystems like plants, soil, water and decaying wood material^36–39^. Therefore, it was not surprising to find these 3 genera on the surfaces of this new winery. These genera can therefore be qualified as ubiquitous genera.

**Table 2:** Percentage of non-enological yeast genera identified on the different WREs throughout 2016, 2017 and 2018 vintages.

Yeast genera	Floor					Walls					Equipment					
	2016		2017		2018	2016		2017		2018	2016			2017		2018
	T0	T3	T0	T3	T0	T0	T3	T0	T3	T0	T0 NE*	T0 UE*	T3	T0	T3	T0
***Buckleyzyma***		2.24	2.97	2.49	1.84	**0.77**	0.18	0.44	0.08	0.59	**1.13**	**0.22**	0.03			0.21
***Cutaneotrichosporon***		0.36	0.10	1.06	0.23	**0.70**	0.09								0.26	0.04
*Cystofilobasidium*				0.03					0.01				0.03			
***Exophiala***	**25.36**	8.45	46.45	31.43	13.09	**2.16**	2.30	15.34	6.67	21.98	**0.19**	**0.05**	0.06	0.12	3.26	5.05
***Hannaella***	**10.43**	6.35	26.44	1.79	20.19	**1.24**	0.60	7.13	7.62	37.36	**0.19**	**0.05**	0.09		0.05	2.49
***Kondoa***												**0.11**				
*Kregervanrija*				0.05	0.03		0.18			0.02					0.53	
*Kwoniella*				0.06									0.03			
***Leucosporidium***	**8.83**	9.68	10.35	5.29	1.85	**4.87**	0.78	16.47	13.44	14.58				1.53	1.74	0.42
*Lodderomyces*				0.07												
*Malassezia*			0.01													
*Oberwinklerozyma*								0.10								
*Occultifur*									0.06							
*Ogataea*				0.11	0.14		0.09		0.04							
*Papiliotrema*			0.02		0.26				0.01				0.75			
***Piskurozyma***	**0.32**		0.04						0.04					0.12		
*Pseudomicrostroma*													0.03			
***Saitozyma***								0.13			**1.31**					
*Schwanniomyces*									0.02							
*Trichosporon*				0.05	0.07		0.23								0.42	
***Udeniomyces***		1.01				**0.31**					**0.38**	**0.05**	0.02	1.76		
***Yamadazyma***	**3.69**	0.72	0.02	0.15	0.33	**0.08**	1.70	0.10		0.02					2.10	0.42
**Total**	**48.64**	**28.81**	**86.42**	**42.57**	**38.04**	**10.12**	**6.16**	**39.71**	**27.98**	**74.55**	**3.19**	**0.49**	**1.07**	**3.53**	**8.36**	**8.63**

Calculation was realized on the overall yeast populations.

Bold type genera correspond to those identified at T0 2016.

*(NE) New Euipment and (UE) Used Equipment.

Between T0 2016 and T0 2018, a significant increase in the biodiversity and quantity of non-enological yeasts were observed. Biodiversity-wise, 21 genera were detected at T0 2018 while only 8 genera were found at T0 2016. Quantity-wise, a 7-fold increase in the percentage of non-enological yeast was observed between these two time points on the walls (Table 2). Transfers of yeasts between different surfaces can be carried out by air flow and bioaerosol activity^40^ and over a period of one year^27^. Thus, these different transfers may explain the increase and dynamics of non-enological yeasts between T0 2016 and T0 2018. The two most abundant genera *Exophiala* and *Hannaella*, found initially at T0 2016, persisted in the winery environment throughout all the vintages and on all the winery surfaces. These two genera presented very high percentages, especially at T0 2017 on winery floor (73%) and T0 2018 on the winery walls (59%), however they did not exceed 5% on the winery equipment (Table 2). So, these results suggest that the conditions of every WRE plays an important role in retaining and shaping the fungal consortium of the winery. Indeed, the proliferation of fungi detected on different building material or in a particular area depends on the growth material and the conditions (e.g. humidity and temperature) under which they are found^40,41^. Compared to *Exophiala* and *Hannaella*, the genus *Leucosporidium* also persisted and colonized all WREs but with low percentages. Other genera previously detected at T0 2016, like, *Buckleyzyma, Cutaneotrichosporon* and *Yamadazyma* were detected frequently on all WREs but at very low levels (highest percentage: 2%) over the three vintages. Eleven new genera (e.g. *Cystofilobasidium, Kondoa, Kregervanrija, Kwoniella, Lodderomyces, Malassezia, Oberwinklerozyma, Occultifur, Ogataea, Papilioterma, Piskurozyma, Pseudomicrostroma, Saitozyma, Schwanniomyces, Trichosporon* and *Udeniomyces*) were detected punctually (1 or 2 times between T0 2016 and T0 2018) and the majority of them were detected after the fermentation activity (Table 2).

As for mold and filamentous fungi, different behaviors of non-enological yeasts were observed: some genera that can be considered as resident (environmental flora) and capable of persisting (*Exophiala* and *Hannaella*), genera capable of establishing and persisting (*Buckleyzyma, Cutaneotrichosporon, Leucosporidium* and *Yamadazyma*) and finally genera brought by the fermentation activity (e.g. *Kregervanrija* and *Ogataea*).

### Diversity and dynamics of wine-associated yeast genera

**Figure 3:**
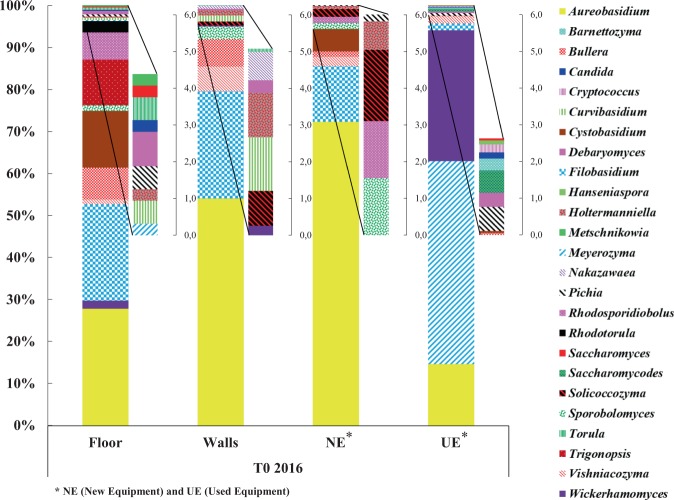
Relative abundance of wine-associated yeast genera identified on the different WREs of the new winery at T0 2016.

At T0 2016, the taxonomic profiles for the floor, walls and the new equipment are quite close compared to the used equipment (Fig. 3), as described previously for mold and filamentous fungi. Relative abundance profiles show that the winery floor, walls and new equipment are primarily dominated by the yeast-like genus *Aureobasidium* (30%, 55% and 70%, respectively) and the genus *Filobasidium* (22%, 29% and 15%, respectively); however the used equipment is dominated by the genera *Meyerozyma* (45%), *Wickerhamomyces* (30%) and *Aureobasidium* (14%) (Fig. 3). Genus *Aureobasidium* was a dominant and common genus to all WREs, probably due to its capacity to persist in stress conditions linked to the sporulation ability of this genus^42^.

Before the arrival of the first harvest, yeast genera of enological interest like *Candida, Hanseniaspora, Pichia* and *Saccharomyces* were detected. Their presence on the used equipment is not surprising and is linked to the presence of must residues and/or wine, particularly in areas that are difficult to clean. The detection of the genera *Candida*, *Pichia* and *Saccharomyces* on the winery floor could be explained by the potential transfer between this environment and the used equipment due to human activities. Before the arrival of the first harvest, none of the yeast genera of enological interest were identified on the walls (Fig. 3).

#### Dynamics of wine-associated yeasts through three vintages on floor

**Figure 4:**
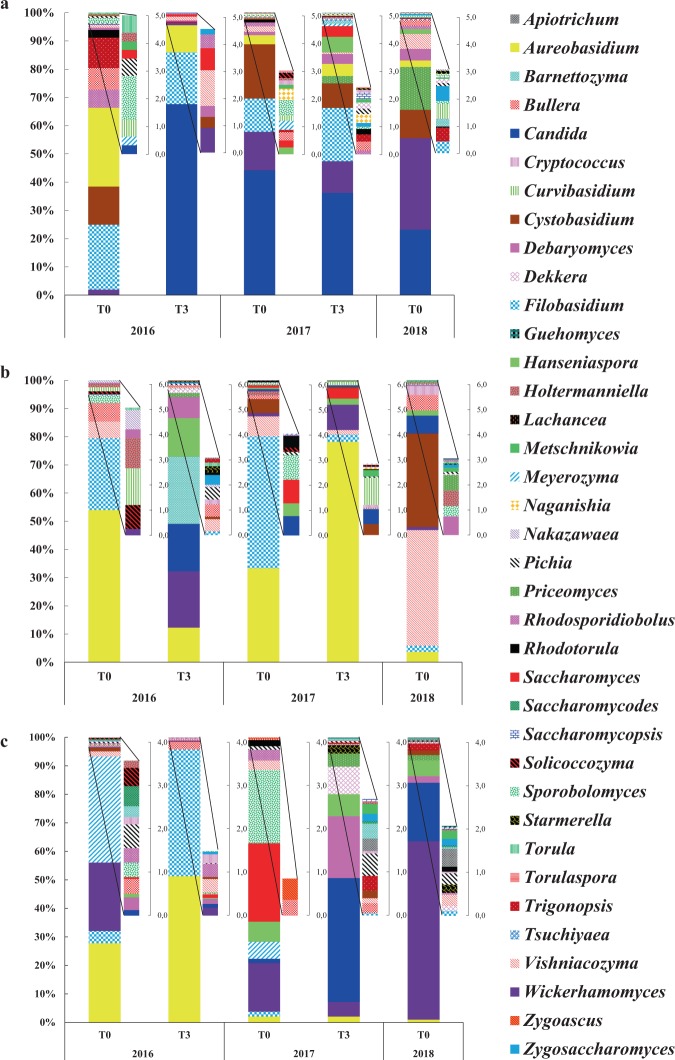
Relative abundance of wine-associated yeast genera identified on winery floor (**a**), walls (**b**) and equipment (**c**) throughout 2016, 2017 and 2018 vintages. Taxonomic profile at T0 2016 is the average of profiles obtained on old and new equipment.

On the winery floor at T0 2016, the 5 genera *Aureobasidium, Candida, Cystobasidium, Filobasidium* and *Wickerhamomyces* represented more than 65% of the total abundance (Fig. 4a). These genera were detected over time with the highest abundance: at T3 2016 (95%), at T0 2017 (92%), at T3 2017 (79%) and at T0 2018 (70%), but also with changes in their percentage of abundance. Indeed, a 75-fold increase of the genus *Candida* was observed at T3 2016, probably due to the fermentation activity (transfer from must and wine). The genera *Bullera, Vishniacozyma* and *Metschnikowia* detected at T0 2016 were also detected over time. The genus *Saccharomyces* detected at T0 2016 (<1%) was detected only at T3 2016 till T3 2017 but not at T0 2018. The genera *Hanseniaspora, Priceomyces* and *Starmerella* described on grapes and must^19,42^ were newly detected probably brought in during the harvest with the grapes and harvest equipment.

During the 3 vintages, the presence of spoilage microorganisms like the genera *Dekkera* (as from T3 2017) and *Zygosaccharomyces* (as from T3 2016) was observed on the winery floor (Fig. 4a). The presence of these yeasts 3 months after the AF could be strongly linked to the fermentation activity (transfer from must and wine). However, their implantation and persistence took place on the winery floor only after the second harvest.

Therefore, we showed that the communities found at T0 2016 conditioned the future resident flora (T0 2018) on the winery floor by the presence over time of the same 16 genera, particularly the genera *Aureobasidium, Candida, Cystobasidium, Filobasidium, Metschnikowia* and *Wickerhamomyces*, while others (*Solicoccozyma* and *Torula*) showed punctual presence. We also showed that the AF might underlie the enrichment (*Candida*) or introduction of genera of interest (*Hanseniaspora* and *Starmerella*) and/or spoilage (*Dekkera* and *Zygosaccharomyces*) yeasts on the winery floor.

#### Dynamics of wine-associated yeasts through three vintages on walls

Some dominant genera on the walls were in common with those found on the floor, like *Aureobasidium, Bullera, Filobasidium, Vishniacozyma* and *Wickerhamomyces*. Their evolutions were similar as they were detected over the three vintages but changes in their percentage of abundance are observed. New genera like *Candida, Cryptococcus*, *Cystobasidium, Hanseniaspora, Pichia, Saccharomyces* and *Starmerella* were detected after the first or the second harvest (Fig. 4b). Their presence on the walls may have been due to transfer *via* grape berries and/or must because they are also commonly described on grape berries and must^26,43^ or to the result of transfers between WREs by air flow or bioaerosol activity. In addition, the increase in the abundance of some genera already present on the walls at T0 2016, such as *Debaryomyces*, may also have been due to fermentation activity and/or to the transfer of these genera between WREs. The spoilage genera *Dekkera* and *Zygosaccharomyces* were detected only at T3 2016 and at T0 2018 (Fig. 4b), and did not show real persistence.

Thus, after 2 years, the resident flora of winery walls included 6 wine-associated yeast genera (*Aureobasidium, Bullera, Debaryomyces, Filobasidium, Vishniacozyma* and *Wickerhamomyces*) found between T0 2016 and T0 2018, as previously described for winery floor. The AF activity also impacted the yeast flora of the walls (particularly the first fermentation) by the enrichment of certain genera like *Debaryomyces* and *Wickerhamomyces* and by the occurrence of new genera like *Candida*, *Cryptococcus*, *Dekkera, Metschnikowia, Pichia*, *Saccharomyces, Starmerella* and *Zygosaccharomyces*. The genus *Saccharomyces* was only detected during the 2017 vintage (T0 and T3), and did not persist in 2018. However, certain genera persisted until 2018 (e.g. *Candida, Hanseniaspora*). In addition, the flora of the walls showed radical changes at different time points. Thus, the resident flora of the walls appeared to have a higher state of instability than that of the winery floor. This instability may also be due to the fact that several genera, mainly of enological interest, were not in an environment favorable for their installation. Their presence appeared to be the result of transfers from other surfaces.

#### Dynamics of wine-associated yeasts through three vintages on equipment

At T0 2016 an average taxonomic profile was established by the association of the 2 profiles obtained from the used and new winery equipment (Fig. 4c). The genera *Aureobasidium* and *Filobasidium*, which were more abundant for the 2016 vintage (as for floor and walls), presented very low abundances afterwards (10% at T0 2017, 2% at T3 2017 and 1% at T0 2018). Throughout the vintages, the taxonomic profiles became enriched by other genera, most importantly, *Candida, Debaryomyces, Hanseniaspora, Metschnikowia, Saccharomyces, Sporobolomyces* and *Wickerhamomyces* which became more widespread (Fig. 4c). The presence of these genera, and more particularly for the genus *Saccharomyces*, probably resulted from must handling (beginning of AF in a fermentation tank) before the sampling of the winery equipment at T0 2017. After this time point, the genus *Saccharomyces* was not detected, so does not seem to persist. The higher presence of non-*Saccharomyces* yeasts compared to *Saccharomyces* on winery equipment have been previously reported^44^.

The spoilage genera *Dekkera* and *Zygosaccharomyces* were both probably provided by must and/or wine, particularly for the 2017 vintage. The highest abundance of the genus *Dekkera* was observed at T3 2017 and persisted to T0 2018 (Fig. 4c).

Thus, the changes of wine-associated yeasts on winery equipment showed specific dynamics compared to the floor and walls. Shifts in the taxonomic profiles were observed between the 2016 vintage and the next vintages. These observations and interpretations must take into account that winery equipment is regularly cleaned and is in direct contact with must and wine. Also, the results show that the evolution of wine-associated yeasts on equipment is not conditioned by the initial yeast population (T0 2016) as found for the winery floor and walls. Despite this, some wine-associated yeast genera appear to be ubiquitous and adapted to winery conditions. Indeed, the genera *Aureobasidium, Bullera, Filobasidium and Vishniacozyma* were detected throughout the whole study no matter which WRE it is.

### Establishment of a winery consortium

The presence of mold, filamentous fungi and yeasts on the WREs of a new winery before the arrival of the first harvest was described. Considering the different abundances of all the genera (not only those which represent >1%), a total of 329 different fungal genera were identified at T0 2016 (294 mold and filamentous fungi and 35 yeasts) on all the WREs, which have never been described before. After 2 years, 172 fungal genera (132 mold and filamentous fungi and 40 yeasts) were identified and 17 fungal genera were newly detected (5 mold and filamentous fungi and 12 yeasts).

**Figure 5:**
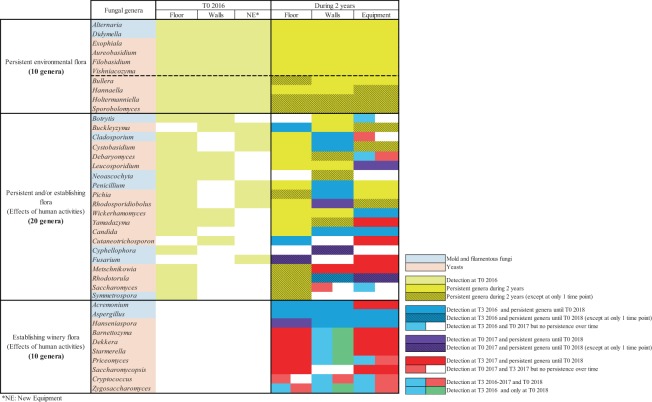
Resident and established fungal genera detected on the different WREs. Mold and filamentous fungi genera represented are >1% of relative abundance.

Among the total fungal genera and in terms of abundance, a total of 40 genera (mold, filamentous fungi and yeasts) presented different evolutions over time (Fig. 5). On the one hand, 10/40 genera (*Alternaria*, *Aureobasidium, Bullera, Didymella, Exophiala, Filobasidium, Hannaella, Holtermanniella, Sporobolomyces* and *Vishniacozyma*) were detected before the first harvest (T0 2016) and throughout the whole study on all the WREs until T0 2018 (Fig. 5). The majority of these 10 genera are commonly described on the berries and/or in must and have been considered, so far, as related to vineyard and wine-growing environments. However, according to the literature, these genera are described as ubiquitous genera widely distributed in natural ecosystems (soil, air, water and plants) and can colonize buildings^45–47^. In fact, the genus *Alternaria* is able to survive in cold, hot and dry weather and all year round in debris and seeds^48–50^. In addition, the sporulation capacity described for mold and fungi genera could contribute toward enhancing their persistence in adverse conditions like the winery environment^34,51^. Certain phenotypic traits described for the persistent genus *Aureobasidium* like the production of extracellular polysaccharides^52^ and film forming capacity^53^ may explain its persistence throughout the study. The persistence of *Bullera*, *Filobasidium* and *Vishniacozyma* genera may be related to their belonging to the Basidiomycetous yeast division and more particularly to the oxidative group of Basidiomycetous. Indeed, this group is described as being usually found in higher dominance in stressful environments and persisting well in adverse environments^54,55^. Several studies demonstrated the tolerance of the genus *Exophiala* to different stresses^56^ and the *Hannaella* yeast genus has been described in the phyllosphere of various plant species including *Vitis vinifera* and in soil^35,57^ which may explain their persistence over time. Thus, according to the results obtained in this study and the specific capacities of the genera described previously, we confirm that these 10 genera are not specific to a given environment like that of wineries. These genera are resident environmental flora that can persist in stressful environments including a winery (Fig. 5).

In addition, 20/40 of the fungal genera were detected at T0 2016 and showed persistence after 2 years on 1 or 2 WREs (Fig. 5). As previously described, ubiquitous character of these fungal genera linked to some phenotypic traits can explain their persistence^37,39,54,55,58–70^. These genera are considered as persistent and/or establishing winery flora. Among them, some genera became established *via* the harvest or transfers between WREs, especially on the walls and equipment (Fig. 5). After their implantation, these genera were able to persist until 2018, except the genus *Saccharomyces*, which persisted only on the floor 2 years after the arrival of the first harvest (Fig. 5). After the implantation linked to harvest, the persistence of the genus *Candida* may have been due to phenotypic traits like invasive growth^71^ and film formation^53^. Regarding the genus *Pichia*, the capacity of some species, isolated from grapes, to form ascospores^72,73^ and to produce killer toxins (proteins secreted by a fungi inhibiting the growth of other fungi)^74^ potentially contribute to the implantation and persistence of this genus in winery environment. Establishment and persistence of the genus *Metschnikowia* may be linked to its ability to outgrow other microorganisms due to the production of reddish pulcherrimin involved in iron chelation^75,76^. Furthermore, among the genus *Wickerhamomyces*, the species *W*. *anomalus* (previously known as *Pichia anomala*)^77^ is known for its resistance to osmotic pressure and the production of killer toxins^78–80^.

Finally, 10/40 of the fungal genera (*Acremonium*, *Aspergillus*, *Barnettozyma*, *Cryptococcus*, *Dekkera*, *Hanseniaspora*, *Priceomyces*, *Saccharomycopsis*, *Starmerella* and *Zygosaccharomyces*) were not detected at T0 2016 but exhibited implantation after the 2016 and/or 2017 harvests and certain of them persisted over time depending on the WREs (Fig. 5). The capacity of the genus *Aspergillus* to produce conidia^81^ and the antagonism activity of the genus *Acremonium*^82^ can explain their establishment in winery environment. Regarding the genus *Hanseniaspora*, the implantation and the persistence of 2 species of *Hanseniaspora* during 2 vintages has been previously demonstrated^19^. The genus *Priceomyces* has never been described in a winery environment before, only on grape berries^43^. Nevertheless, this genus was detected over one year in brewery air^83^. Thus, all yeast genera showed potential for implantation in all WREs and to become part of the established winery flora as well as the spoilage genus *Dekkera* previously described as a persistent genus in several steps of the winemaking process and in WREs^84,85^. So, the implantation of all 10 genera can be explained by human activities (fermentation and/or transfer) in the winery and are considered as establishing winery flora (Fig. 5**)**.

In conclusion, this work provided new insights into the establishment of the fungal consortium in a new winery. This fungal consortium consisted of persistent environmental flora that is not specific to the winery environment. These environmental flora included mold, filamentous fungi (e.g. *Alternaria*, *Didymella*) and yeasts (e.g. *Aureobasidium, Filobasidium*). Certain genera (e.g. *Candida*) were found before the arrival of the first harvest on one WREs, mainly on the winery floor, and persisted over time. Meanwhile, on the other WREs, the same genera were detected following the start of the harvest. Therefore, this finding suggests that these genera are well adapted to one specific environment of the winery and could be classified as persistent flora and/or establishing flora. However, during 2 vintages, the environmental flora was enriched by other fungal genera (e.g. *Acremonium, Hanseniaspora*). These genera were considered as establishing winery flora and are less adapted to the winery environment. Moreover, the majority of establishing flora are genera that present a fermentative metabolism that could also to be a prerequisite state preventing their adaptation. Future works could be carried out to understand the mechanisms implicated in the establishment and persistence of this flora on the WRE of this new winery and more particularly, flora of enological interest.

## Material and Methods

### Facility description

All the samples were collected from a newly established winery located in Nuits-Saint-Georges (Bourgogne, France). The new winery was built in 2016 and started operating in September 2016. The winery covers a surface area of 1000 m^2^ and is situated in an industrial zone with no surrounding vineyards. All the red wines from this winery were obtained exclusively from Pinot Noir grapes.

### Sampling of wine-related environments (WREs)

Samples were taken during three consecutive vintages (2016, 2017 and 2018). For the first vintage, 2016, the samples were collected at two separate time points: before the receipt of grapes (T0), three months after the end of the fermentations (T3), and from three different winery surfaces: floor (F), walls (W), equipment (E). At T0 time point, the majority of the equipment in the new winery was new, except for certain vats (36/108) that were formerly used in another winery. Therefore, the samples collected from the winery equipment (E) at T0 2016 were separated between used equipment (UE) and new equipment (NE). The equipment sampled included lift conveyor belts, destemmers, presses, stainless steel vats, wooden vats, pumps, and transfer pipes.

For the samples collected after T0 2016, no separation between UE and NE was realized since all the equipment was in contact with the must and/or wine and therefore considered as UE.

For the 2017 vintage, the same sampling methodology was followed; however, to obtain more representative samples of the winery, the number was doubled for the samples collected from the floor and walls (Table 3). In the same way, the last samples were taken only at T0 of the 2018 harvest, which means that the sampling was carried out for two full years. All the samples were collected by streaking specific areas of each winery surface using sterile cotton swabs, which were then placed in an equivalent volume (40 mL for 40 samples) of physiological water solution (NaCl at 0.9% w/v).

**Table 3:** Streaking surface and number of samples collected for each winery environment at a sampling time point.

Winery environment		Floor	Walls	Equipment	Total number of samples per time point	Total number of samples /year
Streaking surface		1 000 cm^2^	1 000 cm^2^	4 cm^2^		
Number of samples	2016	40	40	40 (NE) + 40 (UE)*	160	**280 (T0** + **T3)**
	2017	80	80	40	200	**400 (T0** + **T3)**
	2018	80	80	40	200	**200 (T0)**

*NE (New Equipment) and UE (Used Equipment).

A total of 280 samples (2016 vintage), 400 samples (2017 vintage) and 200 samples (T0 2018) were collected on all the winery surfaces (Table 3). Given this significant number, the samples were pooled to obtain average samples per environment and for each sampling time point. For example, for the 2016 vintage, the 40 samples from the floor at T0 were pooled to form an average sample. Thus, a total of 7 average samples (2016 vintage), 6 average samples (2017 vintage) and 3 average samples (T0 2018) were analyzed afterwards.

### DNA extraction

10 mL of each average sample was centrifuged at 4000 rpm for 4 min at 4 °C. The supernatant was discarded and the cell pellet was suspended in 200 µL of DNA-Yeast extraction buffer (2% Triton X-100 (v/v), 1% SDS (w/v), 100 mM NaCl, 10 mM Tris and 1 mM EDTA at pH 8). Then, 60 µL of phenol/chloroform/isoamyl alcohol (25:24:1) and 0.3 g of glass beads (0.5 mm in diameter; Scientific Industries) were added. The cells were lysed by Precellys 24-Dual (Bertin Technologies) for 3 ×45 s and placed on ice for 2 min. Afterwards, 200 µL of TE Buffer was added (10 mM Tris and 1 mM EDTA pH 8) and the mixture was centrifuged at 13 700 rpm for 10 min at 4 °C. The supernatant was collected and the DNA was precipitated with 1 mL of 100% (v/v) ethanol solution and centrifuged at 13 700 rpm for 10 min at 20 °C. The DNA pellet was washed with 70% (v/v) ethanol solution and centrifuged at 13 700 rpm for 5 min at 20 °C. Finally, the DNA pellet was dried at 95 °C for 5 min to remove the excess ethanol and re-suspended in 40 µL of Milli-Q water and stored at −20 °C.

### ITS amplicon library preparation

The nuclear ribosomal internal transcribed spacer 2 (ITS2) region of the fungal DNA was amplified for this analysis using a mono index approach. The fusion primers selected for this study were 5.8S-Fun (5′-CAAGCAGAAGACGGCATACGAGAT-NNNNNNNNNNNN-AGTCAGTCAG-GG-AACTTTYRRCAAYGGATCWCT-3′) and ITS4-Fun (5′-AATGATACGGCGACCACCGAGATCTACAC-TATGGTAATT-AA-AGCCTCCGCTTATTGATATGCTTAART-3′). The fusion primers contained the 24- to 29-bp Illumina sequencing adaptor, the 12-bp Golay barcode (5.8S-Fun exclusively), a 10-bp primer pad, a 2-bp linker, and the 21- to 27-bp core primer (see Supplementary Table S1)^86^.

PCR amplification was carried out in a final volume of 50 μL, including 2 μL DNA template, 0.3 µL of Platinum Taq DNA Polymerase 2.5 U (Invitrogen™, Waltham, MA, USA), 1 × Taq buffer, 20 μM dNTP and 1 μL of each primer 10 µM. The PCR program consisted in denaturing for 5 min at 94 °C, followed by 30 cycles of 30 s at 94 °C, 1 min at 60 °C, 1 min at 72 °C and final extension at 72 °C for 5 min. Finally, the pooled PCR products were size-selected with an Invitrogen® 2% E-Gel, purified using the QIAquick purification kit (Qiagen, Germantown, MD). Their concentration was determined with the Qubit® dsDNA HS Assay Kit (Thermo Fisher Scientifics, USA). The pooled PCR products were sequenced with an Illumina® MiSeq sequencer (2 × 300 cycles) (Illumina, San Diego, CA, USA). 20–25% PhiX control DNA was spiked in the run to add base diversity.

### Sequence analysis

Data from Mi-Seq sequencing were analyzed with PIPITS, an automated pipeline for the detection and differentiation of fungal ITS^87^ that produces Operational Taxonomic Unit (OTUs) abundance tables for each metagenomic sample.

## Supplementary information

Supplementary Table S1.

## Data Availability

All relevant data are available upon request.
