# Supplementary material for: The establishment of a fungal consortium in a new winery
Source: Sci Rep. 2020 May 14;10:7962. doi: 10.1038/s41598-020-64819-2 (PMC7224177; doi:10.1038/s41598-020-64819-2)
Supplement: Supplementary file 1 — Supplementary Table S1. [file 41598_2020_64819_MOESM1_ESM.docx]

The establishment of a fungal consortium in a new winery

Abdo, Hany^1^, Catacchio, Claudia Rita^2^, Ventura, Mario^2^, D’Addabbo, Pietro^2^, Alexandre, Hervé^1^, Guilloux-Bénatier, Michèle^1^ and Rousseaux, Sandrine^1*^

^1^ Univ. Bourgogne Franche-Comté, AgroSup Dijon, PAM UMR A 02.102, F-21000 Dijon, France- *IUVV Equipe VAlMiS, rue Claude Ladrey, BP 27877, 21078 Dijon Cedex, France*

^2^ Department of Biology, University of Bari, Bari 70125, Italy

* Corresponding author: sandrine.rousseaux@u-bourgogne.fr[sandrine.rousseaux@u-bourgogne.fr](mailto:Sandrine.rousseaux@u-bourgogne.fr)

Address: Institut Universitaire de la Vigne et du Vin, rue Claude Ladrey, BP 27877, 21078 Dijon Cedex, France

**Supplementary Information**

**Supplementary Table S1** Fusion primers and Golay barcodes used for Illumina sequencing.
